# Prognostic Model That Predicts Benefits of Adjuvant Radiotherapy in Patients With High Grade Meningioma

**DOI:** 10.3389/fonc.2020.568079

**Published:** 2020-11-10

**Authors:** Daijun Wang, Shuchen Sun, Lingyang Hua, Jiaojiao Deng, Shihai Luan, Haixia Cheng, Qing Xie, Hiroaki Wakimoto, Hongda Zhu, Ye Gong

**Affiliations:** ^1^ Department of Neurosurgery, Huashan Hospital, Shanghai Medical College, Fudan University, Shanghai, China; ^2^ Department of Neuropathology, Huashan Hospital, Fudan University, Shanghai, China; ^3^ Department of Neurosurgery, Massachusetts General Hospital, Harvard Medical School, Boston, MA, United States; ^4^ Department of Critical Care Medicine, Huashan Hospital, Shanghai Medical College, Fudan University, Shanghai, China

**Keywords:** high grade meningioma, prognostic model, radiation, prognosis, atypical meningioma, anaplastic meningioma

## Abstract

**Objective:**

Adjuvant radiotherapy is the main treatment modality for high grade meningioma after surgical resection; however, recurrence and survival outcomes vary. The aim of this study was to create a new “prognostic score” that allows personalized recommendations for post-operative adjuvant radiotherapy in patients with high grade meningioma.

**Methods:**

Clinical data were collected from 115 patients with high grade meningioma treated with surgical resection and adjuvant radiotherapy. A prognostic model was built based on the hazards ratios of independent prognostic factors yielded by multivariate cox proportional analysis. Calibration and discrimination of the prognostic score was evaluated using good of fit test and Harrel’s C index, respectively.

**Results:**

A total of 115 high grade meningioma patients (72 atypical and 43 anaplastic meningiomas) were enrolled. Three factors were independently associated with progression-free survival (PFS): extent of resection (GTR vs. STR), recurrent status (*de novo* vs. recurrent), and Ki-67 labeling index (<5% vs. ≥ 5%). The respective β-coefficients were used to generate the “prognostic score”. The cohort was divided into low-risk and high-risk groups based on the median prognostic score. Good of fit test showed strong calibration (P = 0.7133) and Harrel’s C index 0.766 indicated a strong discrimination capability of the prognostic score. The Harrel’s C index for OS was 0.60.

**Conclusions:**

Our prognostic model using three basic clinical parameters robustly separated high grade meningioma patients who benefit vs. do not benefit from adjuvant radiotherapy. External validation of our model is warranted to help improve patient selection suitable for adjuvant radiotherapy.

## Introduction

Meningioma is one of the most common primary neoplasms arising in the central nervous system (CNS), accounting for about 36.4% of all CNS tumors (1). It has been classified into three grades and fifteen histological subtypes according to the World Health Organization (WHO) 2016 meningioma grading criterion (2). While most of them are benign and slow-growing tumors, higher tumor histological grade is significantly associated with more malignant phenotypes and worse patient outcome, regarding both recurrence and premature mortality. WHO Grade II meningioma was defined by 4–19 mitoses per 10 hpf, brain invasion or presence of the histological features associated with atypia. WHO Grade III meningiomas have a mitotic index higher than 20 per 10 hpf (2). Among high grade meningiomas, atypical (grade II) and anaplastic meningioma (grade III) represent the most common two subtypes. Studies report 5-year survival rates ranging from 78% to 91% and 35% to 79% for atypical and anaplastic meningioma, respectively (3–13).

Although efforts have been made through a dearth of treatment options and biological targets, surgery remains the mainstream treatment strategy (14, 15). Radiation followed by surgical resection is usually recommended for high grade meningioma due to the high rate of recurrence. However, Despite post-operative radiation therapy tumor recurrence or progression is not uncommon, suggesting that adjuvant radiation is only effective in a subset of the patients (9, 16–22). Therefore, patients with high grade meningioma must be appropriately stratified to select patients who are more likely to benefit from adjuvant radiotherapy.

We here propose a novel and simple evaluation score based on basic pre and post-operative clinical information to predict and assess the efficacy of adjuvant radiation therapy regarding both tumor recurrence and overall survival. This scoring model provides us with a clinically applicable tool that assists with personalized treatment recommendations and enables predictions of treatment outcomes in these heterogeneous patients.

## Patients and Methods

### Study Cohort

During the period between January 2003 and December 2008, a total of 115 patients underwent surgical resection of high grade meningioma (atypical and anaplastic) and received post-operative radiation therapy at the Department of Neurosurgery, Huashan Hospital, Fudan University, providing the study cohort for model development and detailed analysis. Patients demographics including age at admission, gender, preoperative Karnofsky performance scale (KPS), tumor location, tumor histological grade, extent of surgical resection, and outcome data were collected and analyzed. The pathological results of all the 115 patients were rechecked and confirmed by two experienced neuro-pathologists (Hong Chen and Yin Wang) according to the 2016 WHO CNS tumor grading criterion. WHO grade II and III meningiomas diagnosed as rarer pathological variants were not included. Meningioma surgical resection was evaluated based on post-operative enhanced T1-weighted MRI and surgical records according to the Simpson grading criterion, and were classified to gross total resection (GTR, Simpson grades I–III) and subtotal resection (STR, Simpson grades IV–V) subgroups. Tumor location was divided into “skull base” and “non-skull base” locations. Follow-up was conducted routinely according to the guidelines of Huashan Neurosurgical Center. Tumor progression was identified as tumor enlargement compared to previous images at the operative location via post-operative MRI. Progression-free survival (PFS) and OS were defined as time since surgical resection to tumor progression or to death as a result of any cause or censored at the date of the last follow-up. Written informed consent was obtained from all patients involved in our study. This clinical study was approved by the Human Subjects Institutional Review Board at Huashan Hospital, Fudan University (KY-2017-09).

### Postoperative Radiotherapy

All patients received post-operative radiotherapy within 2 to 4 weeks after the surgery. Either conventional external beam radiotherapy or Gamma knife was applied. The selection of the type of radiation therapy was based on radiation oncologists’ decision as well as patients’ preference. The planning protocol for radiation therapy was delineated according to the treatment protocol of Huashan Radiation center. For traditional external beam radiation therapy, 2.0 Gy daily fractions with 1- to 2-cm clinical target volume (CTV) and 3- to 5-mm planning target volume (PTV) was applied with the mean total dose 48.9 ± 5.1 Gy (range 32–66 Gy). For Gamma-knife treatment protocol, the prescription dose was 14.0 Gy at 50% and 28.0 Gy at 100%.

### Statistics

Based on previous studies and our own experiences which reported association between clinical indices and outcome of high grade meningioma, we put forth the primary hypothesis that a constellation of clinical and treatment parameters is associated with the efficacy of radiation on patients with high grade meningioma and that a prognostic score based on a weighted model of these parameters will assist decision making whether or not to apply radiation to these high grade meningioma patients. PFS was used as the primary endpoint for model development since tumor recurrence was the most clinically relevant. In addition, the model was validated for its predictability of OS as well. We turned continuous factors such as age and Ki-67 index into dichotomies according to suggestions proposed by P Royston et al. (23). The model development approach was in kept with Transparent Reporting of a Multivariable Prediction Model for Individual Prognosis or Diagnosis guidelines.

### Model Building

Univariate Cox-proportional hazards regression model was initially used to identify prognostic factors for tumor recurrence. Clinical factors considered for prognostic analysis included: age (<60 years vs. ≥60 years), gender (female vs. male), WHO grade (grade II vs. grade III), treatment status (newly diagnosed vs. recurrent), Simpson resection grade (GTR vs. STR), and Ki-67 index (<5% vs. ≥5%). Factors with a *P* value less than 0.05 in the univariate Cox regression model were further included in multivariate Cox proportional hazards regression model for building the prognostic score by using a backward elimination procedure. Non-significant factors (*P* ≥ 0.05 in the multivariable Cox-proportional hazards regression model) were removed from the model with a stepwise procedure. The model fitting was evaluated by using the Akaike information criterion (AIC) and the model with the smallest AIC was selected as the final prognostic score (23). The predictive ability of the model was evaluated by its discrimination and calibration. The discriminative ability was examined with Harrell c-statistics, while calibration was assessed through Hosmer-Lemeshow goodness of fit test as well as comparing the observed and predicted survival rate at 18, 24, 30, 36, 48, 60, and 72 months.

The regression coefficient for each independent prognostic factor was computed from the equation [β = ln (HR)], in which HR is the hazards ratio in the multivariate Cox regression model. The prognostic score was calculated for each patient by the sum of the individual scores. The cohort was dichotomized into low-risk and high-risk subgroups according to the median prognostic score of the whole cohort to predict patients that did not benefit from adjuvant radiotherapy.

Statistical analysis was performed using software STATA 13.3 for windows (StataCorp, College Station, TX). Clinical data such as medians were summarized with descriptive analysis. Categorical variables were compared with either Pearson chi-square test or Fisher’s exact test. Student t test (data with normal distribution) or Mann-Whitney U test (data with skewed distribution) was used for continuous variables. Survival curves were estimated by Kaplan-Meier method. *P* < 0.05 was considered significantly different.

## Results

### Baseline Characteristics

A total of 115 patients with high grade meningioma treated with surgical resection followed by adjuvant radiotherapy at the Neurosurgical center of Huashan Hospital, Fudan University met the inclusion criterion. Among them, 72 (62.6%) were atypical and 43 (37.4%) were anaplastic meningioma. The median age of these patients was 48.05 ± 12.31 years (range: 19–81 years). 59 patients (51.3%) were females. The median of preoperative KPS was 80 (range: 20–100). The most common location in our series was convexity (n = 52, 45.2%), followed by falcine/parasagittal (n = 38, 26.1%) and skull base (n = 25, 21.7%). GTR was achieved in 91 cases (79.1%) and the rest of the patients (n = 24, 20.9%) underwent subtotal resection. Thirty patients (26.1%) had a previous history of surgical meningioma resection, and they were diagnosed as recurrent meningioma. Demographic characteristics are summarized in **Table 1**.

**Table 1:** Baseline characteristics of patients in our cohort.

Characteristics	Overall, No. (%)
Age, years	48.05 ± 12.31
<60 ≥60	81 (70.4%) 34 (29.6%)
Gender	
Male Female	56 (48.7%) 59 (51.3%)
WHO grade	
Grade II (atypical) Grade III (anaplastic)	72 (62.6%) 43 (37.4%)
Tumor location	
Skull base Non-skull base	25 (21.7%) 90 (78.3%)
Recurrent status	
*De novo* Recurrent	85 (73.9%) 30 (26.1%)
Extent of tumor resection	
GTR STR	91 (79.1%) 24 (20.9%)
Preoperative KPS score	
<80 ≥80	40 (34.8%) 75 (65.2%)
Recurrent status	
Primary Recurrent	85 (73.9%) 30 (26.1%)
Ki-67 labeling index	
<5 ≥5	64 (55.7%) 51 (44.3%)

KPS, karnofsky performance score; GTR, gross total resection; STR, sub-total resection.

### Follow-Up and Outcome

**Figure 1:**
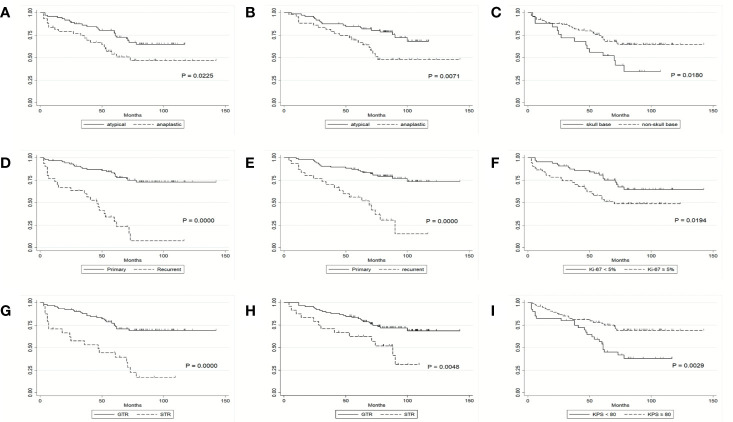
Kaplan-Meier survival curves. **(A)** PFS of patients by histological grade. **(B)** OS of patients by histological grade. **(C)** PFS of patients by tumor location. **(D)** PFS of patients by tumor recurrence status. **(E)** OS of patients by tumor recurrence status. **(F)** PFS of patients by Ki-67 labeling index. **(G)** PFS of patients by extent of tumor resection. **(H)** OS of patients by extent of tumor resection. **(I)** PFS of patients by KPS score.

The median follow-up was 51.8 months (range: 3 to 142 months). Median PFS was 70 months for all patients, with 3-, 5-, and 7-year recurrence free rate being 80.7%, 68.5%, and 57.9%, respectively. For atypical meningioma, median PFS was 71 months, with 3-, 5-, and 7-year recurrence free rate being 86.2%, 77.2%, and 64.8%, respectively. And for anaplastic meningioma, median PFS was 55 months, with 3-, 5-, and 7-year recurrence free rate being 71.8%, 54.0%, and 46.9%, respectively. Median OS for all patients was 77 months, with 3-, 5-, and 7-year survival rate being 85.2%, 78.1%, and 67.6%, respectively. When grouped by tumor grade, median OS was 81 months for atypical meningioma, with 3-, 5-, and 7-year survival rate being 87.5%, 84.7% and 78.4%, respectively. Median OS for anaplastic meningioma was 66 months, with 3-, 5-, and 7-year survival rate being 81.4%, 66.9%, and 47.8%, respectively. There existed a significant difference in both PFS (P = 0.026) and OS (P = 0.009) between grade II and grade III tumors (**Figure 1**).

### Univariate and Multivariable Progression-Free Survival

Clinical factors listed in **Table 2** were firstly tested for their association with PFS using the Cox proportional hazards model; significant prognostic factors for PFS on univariate analysis were histological grade (HR, 1.96; 95% CI, 1.09 to 3.57; P = 0.026), recurrent status (HR, 1.90; 95% CI, 1.3 to 2.77; P = .001), tumor resection grade (HR, 4.01; 95% CI, 2.19 to 7.34; P = 0.000), Ki-67 labeling index (HR, 2.01; 95% CI, 1.10 to 3.65; P = 0.022), preoperative KPS (HR, 0.42; 95% CI, 0.23 to 0.76; P = 0.004) and tumor location (HR, 0.48; 95% CI, 0.26 to 0.90; P = 0.021) (**Figure 1** and **Table 2**). These factors were further included in the multivariable Cox proportional model. We found that extent of tumor resection (HR, 3.32; 95% CI, 1.74 to 6.33; P = 0.000), Ki-67 labeling index (HR, 2.30; 95% CI, 1.23 to 4.29; P = 0.009) and tumor recurrent status (HR, 4.81; 95% CI, 2.48 to 9.31; P = 0.000) were independent predictors of PFS (**Table 2**).

**Table 2:** Univariate and Multivariate Analysis of Prognostic Factors in high grade meningioma patients rerated with adjuvant radiotherapy.

Variable	Univariate Analysis				Multivariate Analysis			
	PFS		OS		PFS		OS	
	p	HR (95% CI)	p	HR (95% CI)	p	HR (95% CI)	p	HR(95% CI)
Age (<60/≥60)	0.515	0.809 (0.428–1.531)	0.552	0.812 (0.409–1.613)				
WHO grade (grade II/III)	0.026^*^	1.968 (1.086–3.566)	0.009^*^	2.347 (1.237–4.454)			0.010^*^	2.452 (1.234 - 4.871)
Gender (female/male)	0.522	1.215 (0.669–2.207)	0.632	0.856 (0.453–1.618)				
Preoperative KPS (<80/≥80)	0.004^*^	0.418 (0.231–0.757)	0.059	0.541 (0.286–1.024)				
Extent of resection (GTR/STR)	0.000^*^	4.011 (2.191–7.343)	0.007^*^	2.538 (1.296–4.971)	0.000^*^	3.322 (1.744–6.330)		
Location (skull base/non-skull base)	0.021^*^	0.480 (0.257–0.895)	0.326	0.696 (0.338–1.434)			0.046^*^	0.454 (0.208 - 0.987)
Ki-67 index (<5%/≥5%)	0.022^*^	2.006 (1.104–3.646)	0.339	1.365 (0.722–2.579)	0.009^*^	2.302 (1.235–4.293)		
*De novo* (no/yes)	0.000^*^	6.145 (3.313–11.401)	0.000^*^	4.670 (2.430–8.972)	0.000^*^	4.809 (2.484–9.312)	0.000^*^	4.607 (2.374 - 8.944)

KPS, karnofsky performance score; PFS, progression-free survival; OS, overall survival; HR, hazard ratio; CI, confidence interval; GTR, gross total resection; STR, sub-total resection; *p < 0.05 considered statistically significant.

### Construction of the Prognostic Model

The model containing these three factors (i.e., extent of resection, Ki-67 labeling index and recurrent status) yielded the smallest AIC number, thus were included in the final model. We then constructed the “prognostic score” by weighing these three independent prognostic factors based on the β-coefficient of the respective log_10_ (HR). The Harrell’s C index of this scoring system was 0.766 (95% CI, 0.692 to 0.839), indicating a strong discriminative ability of the model. In addition, the Hosmer-Lemeshow goodness of fit test also showed a strong calibration of this model (P = 0.7133). The score of the smallest β-coefficient was assigned as 1 and that of the other two factors was accordingly assigned based on the respective β-coefficient. As a result, the score for STR was 1, it was 1.5 for higher Ki-67 labeling index, and the score was 2 for recurrent tumor. The prognostic score for each patient was then calculated based on the sum of weighed numbers of the factors: The prognostic score = 1* [STR = 1 or GTR = 0] + 1.5 * [Ki-67 LI ≥5 = 1 or Ki-67 LI < 5 = 0] + 2 * [recurrent tumor =1 or *de novo* tumor = 0] (**Table 3**).

**Table 3:** Constructed Prognostic score to predict progression-free survival in high grade meningioma patients with adjuvant radiotherapy.

Covariate	β [β = log (HR)]	Score
Extent of resection	1.57	2 * (0/1; GTR = 0, STR = 1)
Ki-67 index	1.19	1.5 * (0/1; <5% = 0, ≥5% = 1)
Recurrent status	0.83	1 * (0/1; primary = 0, recurrent = 1)
Total computed score and risk stratification		
Low risk High risk		<1.5 ≥1.5

HR, hazard ratio.

### Predicting PFS

**Figure 2:**
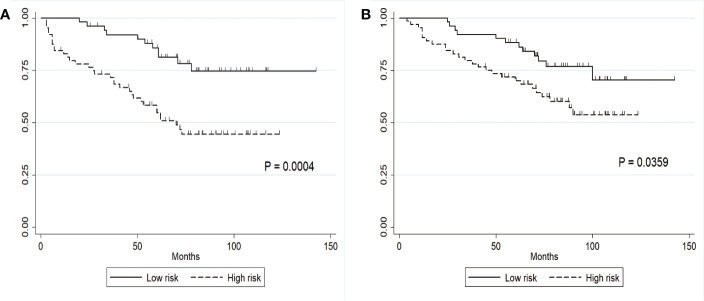
Clinical stratification of PFS and OS on the basis of a constructed prognostic score. **(A)** PFS in the low- and high-risk subgroups defined by a cutoff of 1.5; the cutoff score was the median score in the whole cohort. **(B)** OS in the low- and high-risk subgroups.

**Figure 3:**
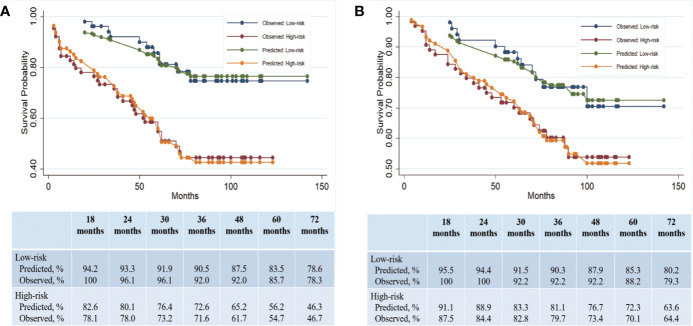
Clinical predication of PFS and OS on the basis of a constructed prognostic score. **(A)** Predicted and observed PFS rates in the low- and high-risk subgroups. **(B)** Predicted and observed OS rates in the low- and high-risk subgroups.

The median prognostic score in our cohort was 1.5 (range: 0–4.5). The score was dichotomized into the low risk and high risk subgroups based on the median cutoff point (i.e., 50^th^ percentile score) of all patients. Fifty-one patients were in the low-risk group and 64 patients were in the high-risk group. The median PFS for the low- and high-risk group was 72 months (range: 20 to 142) and 57 months (range: 1 to 90), respectively, and the difference was significant between the two groups (HR, 2.01; 95% CI, 1.10 to 3.65; P = 0.001, log-rank test) (**Figure 2A**). The Harrell’s C index for this median cutoff point was 0.647 (95% CI, 0.581–0.710). To further validate the predictive accuracy of our prognostic score for PFS in patients with high grade meningioma, the predicted and observed PFS rates at 18, 24, 30, 36, 48, 60, and 72 months of low and high risk subgroups were compared and illustrated in **Figure 3A**. The predicted PFS was closely matched to the corresponding observed probability at these time points.

### Validation of Prognostic Score for OS

The factors that were associated with OS in univariate Cox proportional analysis were histological grade (HR, 2.35; 95% CI, 1.24 to 4.45; P = 0.009), recurrent status (HR, 4.67; 95% CI, 2.43 to 8.97; P = 0.000), and tumor resection grade (HR, 2.54; 95% CI, 1.30 to 4.97; P = 0.007) (**Table 2**). Ki-67 labeling index was not significant (HR, 1.36; 95% CI, 0.72 to 2.58; P = 0.339). In multivariate analysis, the independent factors for OS were tumor recurrent status (HR, 4.61; 95% CI, 2.37 to 8.94; P = 0.000), and tumor grade (HR, 2.45; 95% CI, 1.23 to 4.87; P = 0.010).

Given that the independent factors for OS overlapped with those for PFS, we hypothesized that our prognostic score built with PFS could also serve as a predictor for OS. The median OS in the low- and high-risk groups was 80 months (range: 25–142 months) and 71 months (range: 4–123 months), respectively (**Figure 2B**). The Harrell’s C index of this prognostic score for OS was 0.676 (95% CI, 0.586 to 0.768), indicating a strong discriminative ability of the model. Hosmer-Lemeshow goodness of fit test also showed a strong calibration of this model for predicting OS (P = 0.2657). The OS difference between low- and high groups was significant (HR, 2.05; 95% CI, 1.03 to 4.06; P = 0.04). The Harrell’s C index for this median cutoff point was 0.60 (95%CI, 0.52–0.67). The prognostic score was further evaluated for its calibration by plotting the predicted OS at the mentioned time points, which was also quite close to the observed survival probability at these time points (**Figure 3B**). These results confirmed the strong predictability of our prognostic score for both PFS and OS.

## Discussion

In patients with high grade meningioma after surgical resection, adjuvant radiotherapy is usually recommended to improve PFS and OS. Radiation has been shown to provide significant disease control and longer survival for high grade meningiomas that did not undergo radical resection (5, 8, 9, 24). Based on current reports, the 5-year recurrence free survival rate after adjuvant radiotherapy for grade II meningioma ranges from 48% to 68%, while in grade III meningioma, it drops to 8%–61%, which is quite consistent with our results (16, 17, 20, 25–28). Our series showed a 5-year recurrence free survival rate of 77.2% for atypical and 54.0% for anaplastic meningioma. Although adjuvant radiotherapy is generally thought to be effective for patients with high grade meningioma, grade I evidence is still lacking (29). In addition, a significant proportional of patients do not gain outcomes benefits from adjuvant radiation. Thus, a reliable method of identifying individuals who are more likely to benefit from adjuvant radiotherapy should help select patients with high grade meningioma appropriate for post-operative radiation therapy and avoid overtreatment in unfavorable patients. In our study, patients with the most representative two subtypes of high grade meningioma (atypical and anaplastic) were enrolled for survival analysis. We present a new prognosis scoring system that is based on optimized selection of conventional clinical parameters and is valid in predicting both PFS and OS. Because of the typically long natural history of meningioma, tumor recurrence is clinically relevant, and PFS is the preferred primary clinical endpoint over OS.

In the present study, we evaluated a variety of clinical factors of high grade meningioma treated with adjuvant radiation therapy and identified those that are of prognostic significance. Prognostic factors reported to be predictive of worse survival in high grade meningioma include skull base location, higher tumor grade and less radical resection (4, 5, 7, 8, 30). Some studies reported that lower pre-operative KPS, higher Ki-67 labeling index, tumor recurrent status and estrogen receptor (ER) are also associated with poorer survival (9, 10). However, for patients who received adjuvant radiotherapy, studies about the prognostic factors are limited. Here, we identified that recurrent tumor, higher Ki-67 labeling index, Simpson resection grade, skull base location, preoperative KPS and higher histological grade were associated with worse recurrence free survival. However only tumor recurrent status (*de novo* vs. recurrent), Ki-67 labeling index (<5 vs. ≥5) and Simpson resection scale (GTR vs. STR) were with independent prognostic significance, thus were incorporated to calculate the weighted prognostic score. Ki-67 labeling index is a well-known proliferative indictor in tumors, which is reported to be associated with higher tumor grade and more malignant phenotypes. Our previous study of 87 patients with grade III meningioma also showed significant association of the Ki-67 index with PFS or OS (9). Abry E et al. reviewed a total of 53 publications and found that Ki-67 labeling index can be used as a useful predictor of tumor recurrence in high grade meningioma as well, which was in agreement with our analysis (31).

Since Simpson grade was first asserted in evaluating the extent of resection in 1957, subsequent studies consistently showed that more radical resection was associated with lower rate of recurrence and longer survival. In our previous study of grade III meningioma, more radical resection was associated with longer PFS (9). However, the role of Simpson resection grade in outcomes remains undetermined for patients who receive adjuvant radiotherapy. Recently, Kim D et al. reported that Simpson resection grade was not associated with recurrence risk in their analysis of 76 patients with high grade meningioma treated with adjuvant radiotherapy after surgical resection (10). In contrast, in our cohort, Simpson resection grade was associated with both PFS and OS and served as an independent prognostic factor for PFS. The difference between Kim et al. and us may be caused by factors such as neurosurgeon’s estimate of the degree of resection and use of early postoperative MRI.

Consistent with with our study, several studies have underlined the longer survival in *de novo* high grade meningiomas compared to secondary or recurrent tumors (12, 32, 33). In our previous series of grade III meningioma, we have demonstrated that recurrent tumor, especially those with malignant transformation, tended to have worse outcome (9). Peyre et al. analyzed a series of 57 anaplastic meningioma and suggested different histo-molecular prognostic factors for *de novo* and recurrent tumors, including *TERT* mutation (34), which was further validated by that secondary meningioma had a higher proportion of *TERT* promoter mutation and is associated with significantly worse outcome (35, 36).

Our prognostic model divided the cohort into low-risk and high-risk groups, which had contrasting prognoses regarding both PFS and OS. Since all these three factors are obtained either immediately or days after surgical resection in routine clinical practice, our prognostic score enables clinical stratification and treatment recommendation (radiation vs. no radiation).

### Limitations

Our study is a single institution, retrospective analysis. In order to gain a long-term follow-up result, only patients treated between 2003 and 2008 were enrolled, which limited the sample size. The significance of this study could be reinforced by analyzing a separate validation cohort.

### Conclusions

To the best of our knowledge, this is the first prognostic model for risk stratification in patients with high grade meningioma who were treated with adjuvant radiotherapy. Our prognostic score is robust in predicting both PFS and OS of these individuals and therefore serves as a treatment decision making tool for both neurosurgeons and patients. Our work demonstrates that adjuvant radiation therapy can be a suitable approach for low risk groups but may not be appropriate for some high risk patients. Future work is warranted to adjust our model to improve prediction accuracy.

## Data Availability Statement

The raw data supporting the conclusions of this article will be made available by the authors, without undue reservation.

## Ethics Statement

Written informed consent was obtained from all patients involved in our study. This clinical study was approved by the Human Subjects Institutional Review Board at Huashan Hospital, Fudan University (KY-2017-09).

## Author Contributions

Study concepts: DW, SS, LH, and YG. Study design: DW. Data acquisition: DW, LH, HZ, JD, and SS. Quality control of data and algorithms: HC and HZ. Data analysis and interpretation: LH, DW, HW, and QX. Statistical analysis: LH, DW, and HW. Manuscript preparation: DW, SS, and LH. Manuscript editing: LH, HW, and QX. Manuscript review: HW, YG, and QX. All authors contributed to the article and approved the submitted version.

## Funding

This study was supported by grants from the National Key R&D Program of China (2018YFC1312600 and 2018YFC1312604 to YG), National Natural Science Foundation of China (81772674 to YG), and the Science and Technology Commission of Shanghai Municipality (18140900200 to YG).

## Conflict of Interest

The authors declare that the research was conducted in the absence of any commercial or financial relationships that could be construed as a potential conflict of interest.

## Abbreviations

PFS, progression-free survival; OS, overall survival; CNS, central nervous system; WHO, World Health Organization; KPS, karnofsky performance score; GTR, gross total resection; STR, subtotal resection; CTV, clinical target volume; ER, estrogen receptor.
